# Autocrine VEGF signaling promotes cell proliferation through a PLC-dependent pathway and modulates Apatinib treatment efficacy in gastric cancer

**DOI:** 10.18632/oncotarget.14467

**Published:** 2017-01-03

**Authors:** Yi Lin, Ertao Zhai, Bing Liao, Lixia Xu, Xinhua Zhang, Sui Peng, Yulong He, Shirong Cai, Zhirong Zeng, Minhu Chen

**Affiliations:** ^1^ Department of Gastroenterology and Hepatology, The First Affiliated Hospital of Sun Yat-sen University, Guangzhou, Guangdong, P.R.China; ^2^ Department of Gastrointestinal Surgery, The First Affiliated Hospital of Sun Yat-sen University, Guangzhou, Guangdong, P.R.China; ^3^ Department of Pathology, The First Affiliated Hospital of Sun Yat-sen University, Guangzhou, Guangdong, P.R.China

**Keywords:** autocrine, VEGF, proliferation, Apatinib, gastric cancer

## Abstract

**Background:**

Tumor cells produce vascular endothelial growth factor (VEGF) which interact with the membrane or cytoplasmic VEGF receptors (VEGFRs) to promote cell growth in an angiogenesis-independent fashion. Apatinib, a highly selective VEGFR2 inhibitor, is the only effective drug for patients with terminal gastric cancer (GC) who have no other chemotherapeutic options. However, its treatment efficacy is still controversy and the mechanism behind remains undetermined. In this study, we aimed to investigate the role of autocrine VEGF signaling in the growth of gastric cancer cells and the efficacy of Apatinib treatment.

**Methods:**

The expression of phosphor VEGFR2 in gastric cancer cell lines was determined by real-time PCR, immunofluorescence, and Western blot. The gastric cancer cells were administrated with or without recombination human VEGF (rhVEGF), VEGFR2 neutralizing antibody, U73122, SU1498, and Apatinib. The nude mice were used for xenograft tumor model.

**Results:**

we found that autocrine VEGF induced high VEGFR2-expression, promoted phosphorylation of VEGFR2, and further enhanced internalization of pVEGFR2 in gastric cancer cells. The autocrine VEGF was self-sustained through increasing VEGF mRNA and protein expression. It exerted pro-proliferative effect through a PLC-ERK1/2 dependent pathway. Furthermore, we demonstrated that in VEGFR2 overexpressing gastric cancer cells, Apatinib inhibited cell proliferation *in vitro* and delayed xenograft tumor growth *in vivo*. However, these effects were not observed in VEGFR2 low expressing gastric cancer cells.

**Conclusion:**

These results suggested that autocrine VEGF signaling promotes gastric cancer cell proliferation and enhances Apatinib treatment outcome in VEGFR2 overexpression gastric cancer cells both *in vitro* and *in vivo*. This study would enable better stratification of gastric cancer patients for clinical treatment decision.

## BACKGROUND

Gastric cancer (GC) is the fourth most common carcinoma and the second leading cause of cancer-related mortality worldwide [1]. It is estimated that there are approximately 400,000 new cases in China annually, comprising about 43% globally [2]. Despite advances in chemotherapy and surgery, the prognosis of patients with advanced gastric cancer remains poor [3]. For instance, the 5-year survival rate is only 17.0% for stage IIIC gastric cancer [4]. Therefore, novel chemotherapeutic strategies are needed to treat this lethal tumor.

Angiogenesis is important in some physiological processes, including cell development, wound healing and pathological processes, especially carcinogenesis [5–7]. Angiogenesis is regulated markedly by signaling through vascular endothelial growth factor (VEGF) and its receptors, VEGFR1 (Flt-1), VEGFR2 (KDR) and VEGFR3 (Flt-4) [8]. Tumor cells produce VEGF, which binds with VEGFRs on the stromal, endothelial and tumor cells [9–10]. The interaction between VEGF and VEGFRs results in the recruitment of endothelial progenitor cells to the region surrounding the tumor mass [11–12]. The resultant neovascularization supplies nutrient to support tumor proliferation, growth, and metastasis. Tumor angiogenesis is one of the hallmarks of cancer progress. Therefore, inhibition of VEGF signaling has become an attractive anti-cancer approach.

Angiogenesis inhibitors (AIs) have been hailed as the beginning of a new era in cancer therapy. Some strategies targeting VEGF signaling pathway have been developed, which include neutralizing antibodies to VEGF or VEGFRs, soluble VEGFR/VEGFR hybrids and small molecule VEGFR inhibitors [13]. Bevacizumab, the first drug that inhibits VEGF signaling to be approved by the FDA of the USA for cancer treatment, is a monoclonal neutralizing antibody targeting VEGF [14]. CDP791 and IMC-1121B both are humanized monoclonal antibodies, could directly bind to the extracellular domain of VEGFR2 [15]. Aflibercept (VEGF Trap) is a recombinant fusion protein of the human VEGFR1 and VEGFR2 extracellular domains and the Fc portion of human immunoglobulin G1 (IgG1) [16]. Sorafenib and Sunitinib are multikinase inhibitors with antiangiogenic and antitumor properties that target VEGFRs and other kinases [17–18]. Although these inhibitors could prolong the survival time of tumor patients to a certain extent, the side effect of drugs had adversely influences patient's quality of life.

Apatinib is an oral tyrosine kinase inhibitor (TKI) of VEGFR2 that has anti-cancer activity in some solid tumors [19]. Some studies have confirmed that Apatinib was a more selective inhibitor of VEGFR2 than Sunitinib and Sorafenib, with a 10 times binding affinity of Vatalanib and Sorafenib [20]. Apatinib exhibited objective efficacy in heavily pretreated, metastatic non-triple-negative breast cancer with manageable toxicity, and it was a better choice to be used in breast cancer with high angiogenesis dependency [21–22]. In a phase III clinical trial, Apatinib has been proven to be the only effective pharmacy in the treatment of patients with terminal gastric cancer who do not have other chemotherapeutic options [20]. Although Apatinib has been confirmed effectively in the treatment of solid tumors, our knowledge about the molecular mechanism of the drug action remained obscure.

While the effects of VEGF on endothelial and stromal cells in angiogenesis is well known, some studies suggest that autocrine VEGF signaling in cancer cells plays an important role in affecting cell proliferation and apoptosis [23–24]. Zhang et al [9] and Peng et al [25] confirmed that autocrine VEGF signaling could promote malignant cell proliferation. However, the autocrine VEGF signaling on GC has not been investigated. In this study, we investigated the role of autocrine VEGF signaling on cell proliferation in gastric cancer cells and explored how autocrine VEGF signaling modulates Apatinib efficacy in the treatment of GC.

## RESULTS

### Differential expression of VEGF, pVEGFR2, and VEGFR2 in gastric cancer cell lines

**Figure 1:**
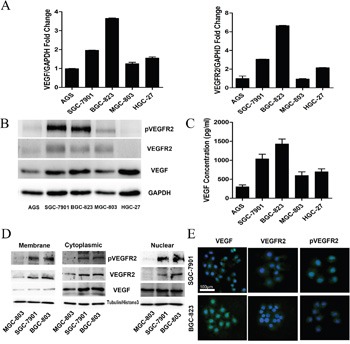
Differential Expression of VEGF, pVEGFR2, and VEGFR2 in gastric cancer cell lines **A**. Expression of VEGF, VEGFR2 was analyzed by qRT-PCR in 5 gastric cancer cell lines. **B**. Expression VEGFR2, pVEGFR2, VEGF protein was analyzed by Western blot in 5 gastric cancer cell lines. **C**. ELISA analysis of the secretion of VEGF in gastric cancer cell lines. **D**. Expression of VEGFR2, pVEGFR2, and VEGF in the cell membrane, cytoplasm, and nucleus of SGC-7901, BGC-823, and MGC-803 cell lines. **E**. Expression of VEGFR2 and pVEGFR2 was analyzed by IF in SGC-7901 and BGC-823 cells.

Some studies indicated that proteins on VEGF signaling pathway were differentially expressed in cancer cells. To determine these expression in gastric cancer cell lines, we firstly detected the mRNA levels of VEGF (Figure 1A, left panel) and VEGFR2 (Figure 1A, right panel) in 5 gastric cancer cells (AGS, SGC-7901, BGC-823, MGC-803, and HGC-27). We found that VEGF and VEGFR2 mRNA levels were significantly higher in SGC-7901 and BGC-823 than other cell lines. Then, we detected the protein level of VEGF, pVEGFR2 and VEGFR2 in these gastric cancer cell lines. The activated VEGFR2, phosphorylation of VEGFR2 (pVEGFR2), were differently expressed, while the higher level in SGC-7901 and BGC-823, and the lower level in MGC-803, AGS, and HGC-27 (Figure 1B). The mRNA and protein level of VEGFR1 were similarly with VEGR2 in all the cell lines (data not shown). As Apatinib is a highly selective VEGF receptor 2 inhibitor which has little affinity to VEGFR1, we further focus on investigating the role of VEGFR 2 in gastric cancer cell proliferation.

Since secretion of VEGF is required for autocrine signaling, we assessed the amount of VEGF protein secreted in cultured medium by ELISA assay. Gastric cancer cells could secrete VEGF into the medium, especially the BGC-823 and SGC-7901 cell lines (Figure 1C). According to the level of VEGFR2 and VEGF, BGC-823, SGC-7901 and MGC-803 were chosen for investigating the mechanism of autocrine VEGF signaling in cell proliferation in gastric cancer.

As a receptor of VEGF, VEGFR2 located on cell membrane; however, some studies indicated that VEGFR2 could translocate into intracellular [24, 27]. To detect the expression levels of VEGF and VEGFR2 in the membrane, cytoplasm and nuclei, we performed Western blot with fractionated membrane, cytoplasm and nuclear proteins from SGC-7901, BGC-823 and MGC-803. The data demonstrated that VEGFR2 was mostly located in the cytoplasm and membrane, whereas the activated form, pVEGFR2, was observed primarily in the nucleus and cytoplasm (Figure 1D). Furthermore, the location of VEGFR2 and pVEGFR2 was confirmed by immunofluorescent staining in SGC-7901 and BGC-823, which have higher activated VEGFR2 expression. The data demonstrated that VEGFR2 was mostly located in the cytoplasm and membrane, whereas the activated form, phosphorylation of VEGFR2 was observed primarily in the nucleus and cytoplasm (Figure 1E).

### Inhibition of VEGF-VEGFR2 signaling decreased cell proliferation and VEGF secretion in gastric cancer cell lines

**Figure 2:**
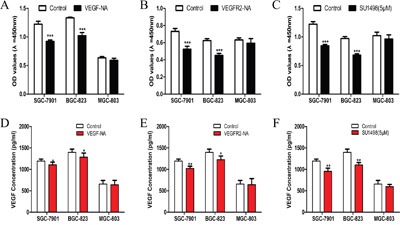
Inhibition of VEGF-VEGFR2 signaling decreased cell proliferation and VEGF secretion in gastric cancer cell lines **A**. Proliferation of gastric cancer cells in response to VEGF-neutralization antibodies (VEGF-NA) in SGC-7901, BGC-823, and MGC-803 cells. **B**. The proliferation of gastric cancer cells in response to VEGF receptor 2 neutralization antibodies (VEGFR2-NA) in SGC-7901, BGC-823, and MGC-803 cells. **C**. The proliferation of gastric cancer cells in response to SU1498 in SGC-7901, BGC-823 and MGC-803 cells. **D**. VEGF secretion of gastric cancer cells in response to VEGF-neutralization antibodies (VEGF-NA) in SGC-7901, BGC-823, and MGC-803 cells. **E**. VEGF secretion of gastric cancer cells in response to VEGF receptor 2 neutralization antibodies (VEGFR2-NA) in SGC-7901, BGC-823, and MGC-803 cells. **F**. VEGF secretion of gastric cancer cells in response to SU1498 in SGC-7901, BGC-823 and MGC-803 cells. Mean±SEM, t-test, *P<0.05, **P<0.01, ***P<0.001.

To explore the mechanism underlying the growth suppressive effects of VEGF-VEGFR2 signaling inhibition, we studied cell proliferation and VEGF secretion. VEGF-NA, VEGFR2-NA and SU1498 (inhibitor of VEGFR2) were used to block VEGF-VEGFR2 signaling by neutralizing VEGF in extracellular, blocking extracellular or intracellular fragment of VEGFR2. We found that these inhibitors had no significant effect on cell proliferation in MGC-803 cells which with lower pVEGFR2 expression (Figure 2C), but significantly decreased cell viability in SGC-7901 (Figure 2A) and BGC-823 (Figure 2B), which have higher pVEGFR2 expression. By detecting VEGF protein level in cultural medium, compare to SGC-7901 (Figure 2D) and BGC-823 (Figure 2E), although VEGF protein level decreased in MGC-803 (Figure 2F), it did not show statistical significance. This demonstrated that inhibition of VEGF-VEGFR2 signaling decreases secretion of its own ligand. These findings suggested that VEGF secretion by gastric cancer cells may contribute to cell proliferation by binding to VEGFR2 in an autocrine manner.

### Exogenous VEGF promoted pVEGFR2 nuclear translocation

**Figure 3:**
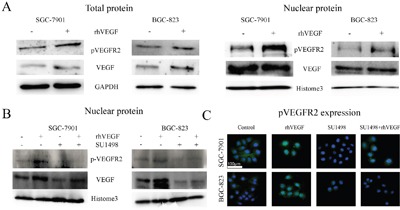
Exogenous VEGF promoted pVEGFR2 nuclear translocation **A**. Expression of pVEGFR2 and VEGF after treating cells with rhVEGF in total protein (left panel) and in nuclear protein (right panel). **B**. Blocking VEGFR2 by SU1498, the expression of pVEGFR2 and VEGF in nuclear protein. **C**. Blocking VEGFR2 by SU1498, the expression of pVEGFR2 were measured by IF.

Some studies demonstrated that activated VEGFR2 could translocate into the nucleus and acted as a transcription factor to modulate cell function [24, 27]. To investigate whether this phenomenon also exists in gastric cancer cells, SGC-7901, and BGC-823 cells were treated with rhVEGF. Western blot and immunofluorescence (IF) were performed to observe the localization of pVEGFR2. After the administration with rhVEGF, the nuclear expression of pVEGFR2 increased in SGC-7901 and BGC-823 (Figure 3A). In order to explore whether blocking VEGFR2 could inhibit pVEGFR2 nuclear translocation, cells were firstly administrated with SU1498 which is a small molecular inhibitor of VEGFR2, following the administration of rhVEGF. We found that nuclear translocation of pVEGFR2 was suppressed by SU1498. However, rhVEGF could not reverse the effects (Figure 3B). These results were consistent with what we found by IF staining in SGC-7901 and BGC-823 cell lines (Figure 3C).

### Autocrine VEGF signaling promoted cell proliferation and its own production in gastric cancer cells

**Figure 4:**
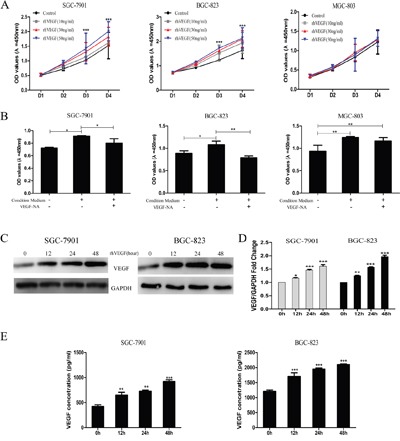
Autocrine VEGF signaling promoted cell proliferation and its own production in gastric cancer cells **A**. The viability of gastric cancer cells in response to recombinant human VEGF (rhVEGF) in SGC-7901 (left panel), BGC-823 (middle panel), and MGC-803 cells (right panel). **B**. Proliferation of gastric cancer cells under basal medium (BM) and condition medium (CM) with or without VEGF-NA in SGC-7901 (left panel), BGC-823 (middle panel), and MGC-803 cells (right panel). **C**. Autocrine VEGF signaling affected gastric cancer cells self-sustained protein level. **D**. Autocrine VEGF signaling affected gastric cancer cells self-sustained mRNA level. **E**. Autocrine VEGF signaling promoted self-secretion in SGC-7901 and BGC-823 cells. Mean±SEM, t-test, *P<0.05, **P<0.01, ***P<0.001.

To determine whether the increased expression of VEGF and activated VEGFR2 are positively associated with cell proliferation in gastric cancer cells, we treated cells with recombination human VEGF (rhVEGF) to characterize the VEGF-mediated cell proliferation. By administrating SGC-7901, BGC-823, and MGC-803 cells with rhVEGF, the cell viability was significantly increased in BGC-823 (Figure 4A, middle panel) and SGC-7901 (Figure 4A, left panel) in a dose- and time-dependent manner, but not in MGC-803 (Figure 4A, right panel). On the base that gastric cancer cells overexpressing VEGFR2 were responsive to rhVEGF treatment, we next investigated whether the physiological concentration of VEGF could promote gastric cancer cell proliferation. We incubated cells with condition medium (CM), which contains the physiological concentration of VEGF, with the presence or absence of VEGF-neutralizing antibody. The cell proliferation of SGC-7901 (Figure 4B, left panel) and BGC-823 (Figure 4B, middle panel) in its CM was significantly higher than those in basal medium (BM), which was obviously revised by VEGF-NA. On the other hand, the cell proliferation of MGC-803 was not enhanced by its CM (Figure 4B, right panel). To elucidate whether autocrine VEGF signaling initiated the self-sustainable cell growth, we measured VEGF expression following the rhVEGF stimulation in cells with higher activated VEGFR2 expression and found that by triggering cells with rhVEGF, the expression of VEGF increased at 12 hours and reached the peak at 48 hours (Figure 4C). We further verified these findings by qRT-PCR assay (Figure 4D) and ELISA assay (Figure 4E).

### Autocrine VEGF signaling promoted cell proliferation through a VEGFR2-PLCγ1-ERK1/2 pathway in GC

**Figure 5:**
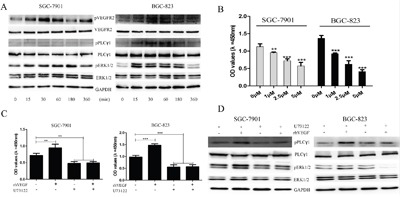
Autocrine VEGF signaling promoted cell proliferation through a VEGFR2-PLCγ1-ERK1/2 pathway in GC **A**. gastric cancer cells were treated with rhVEGF and were harvested at different time points. The time course changes of the phosphorylation of VEGFR2, PLC, and ERK1/2 were detected by Western blot. **B**. The proliferation of gastric cancer cells in response to PLCγ1 inhibitor (U73122) in SGC-7901 and BGC-823 cells. **C**. Blocking PLCγ1 with U73122, proliferation of gastric cancer cells in response to rhVEGF in in SGC-7901 and BGC-823 cells. **D**. After treating cells with U73122, the protein levels were measured by Western blot. GAPDH was included as a loading control. Mean±SEM, t-test, *P<0.05, **P<0.01, ***P<0.001.

Given that VEGFR2 activity was associated with gastric cancer cell proliferation, we further investigated the underlying mechanism *in vitro*. It has been demonstrated that autocrine VEGF signaling promotes cell proliferation through a Phosphoinositide phospholipase C γ1 (PLCγ1) -dependent pathway in hepatocellular carcinoma cells and neoplastic Barrett's epithelial cells [9, 25]. However, whether this effect existed in GC has not been investigated. In this study, we selected highly VEGF-expressed and VEGFR2-actived SGC-7901 and BGC-823 cells for investigating this phenomenon. SGC-7901 and BGC-823 cells were treated with rhVEGF and the potential signaling molecules were examined at different time intervals. The expression of pVEGFR2 increased at 15 min after rhVEGF treatment in SGC-7901 and BGC-803 cell lines and maintained at a high level in 30 min and 60 min. Increased levels of phosphorylate-PLCγ1 and extracellular signal-regulated kinase 1/2 (ERK1/2) were also detected in SGC-7901 and BGC-803 cell lines. We found that PLCγ1 and ERK1/2 were activated after the treatment of rhVEGF (Figure 5A). These results suggested that VEGFR2 signals activated at least partially through PLCγ1 and ERK1/2 pathways in gastric cancer cells.

To further investigate the role of PLCγ1 in gastric cancer cell proliferation, we treated gastric cancer cells with a PLCγ1 inhibitor, U73122, to observe if it will affect gastric cancer cell viability. Compared to the control group (Dimethyl Sulphoxide (DMSO) used only), U73122 significantly decreased SGC-7901 and BGC-823 cell proliferation at the concentrations of 1μM, 2.5μM, and 5μM (Figure 5B), and rhVEGF could not reverse the inhibitory effect of U73122 (Figure 5C). To confirm whether U73122 could inhibit the activation of PLCγ1 and ERK1/2 which induced by rhVEGF, cells were treated with DMSO or 5μM of U73122 overnight, followed by 30min exposure to rhVEGF. Again, without U73122, rhVEGF induced the phosphorylation of PLCγ1 and ERK1/2 in SGC-7901 and BGC-823 cells. However, administration with U73122 diminished the rhVEGF induced phosphorylated PLCγ1 and ERK1/2 to a sub-baseline level (Figure 5D). These findings further suggested that autocrine VEGF signaling can promote cell proliferation in a PLCγ1-dependent fashion.

### Inhibition of VEGFR2 by Apatinib decreased cell proliferation by blocking VEGFR2-PLCγ1-ERK1/2 pathway and reduced VEGF secretion

**Figure 6:**
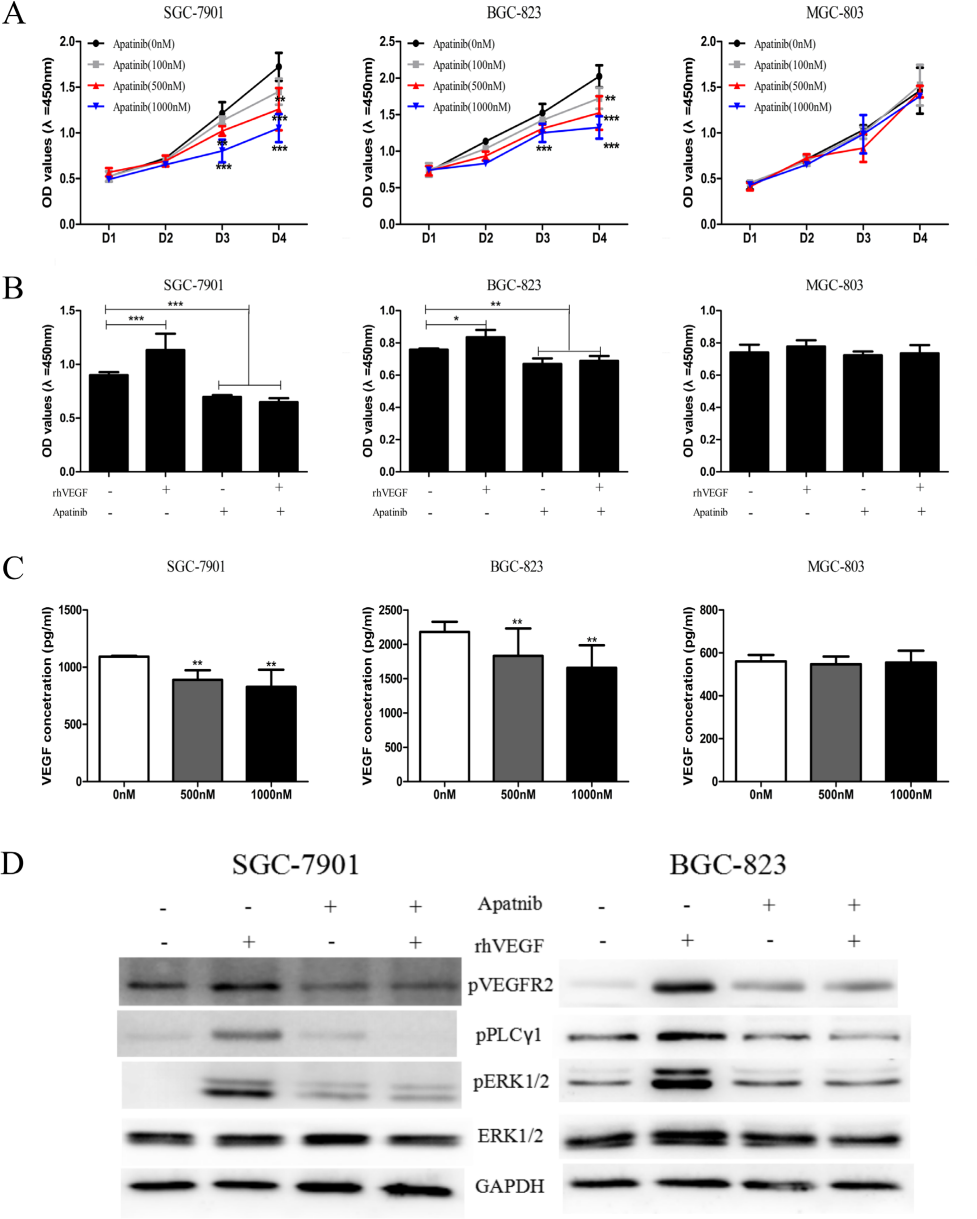
Inhibition of VEGFR2 by Apatinib decreased cell proliferation by blocking VEGFR2-PLCγ1-ERK1/2 pathway and reduced VEGF secretion **A**. The viability of gastric cancer cells in response to Apatinib in SGC-7901 (left panel), BGC-823 (middle panel), and MGC-803 cells (right panel). **B**. Treating with Apatinib, proliferation of gastric cancer cell lines in response to rhVEGF in SGC-7901 (left panel), BGC-823 (middle panel), and MGC-803 cells (right panel). **C**. Treating cells with Apatinib at different concentration affected secretion of VEGF in SGC-7901 (left panel), BGC-823 (middle panel), and MGC-803 cells (right panel). **D**. The protein levels were measured by Western blot after treating cells with Apatinib. GAPDH was included as a loading control. Mean±SEM, t-test, *P<0.05, **P<0.01, ***P<0.001.

Apatinib, a small molecule which targets VEGFR2, has been recommended as the third-line treatment for gastric cancer patients [20], while the mechanism how Apatinib suppresses tumor progression is obscure. To explore whether the autocrine VEGF signaling plays a role on the treatment effect of Apatinib, we investigated the effect of Apatinib on cell proliferation, VEGF secretion, and the VEGFR2-PLCγ1-ERK1/2 signaling pathway. The data showed that treatment with 100 nM, 500 nM and 1000 nM of Apatinib significantly suppressed cell proliferation in SGC-7901 (Figure 6A, left panel) and BGC-823 cells (Figure 6A, middle panel) that overexpressed VEGFR2, pVEGFR2, and VEGF. Treatment with rhVEGF did not reverse Apatinib's inhibitory effect (Figure 6B) in these cells. However, under the same concentration of Apatinib, cell proliferation was not suppressed in MGC-803 cells which had lower expression of VEGF signaling pathway (Figure 6A right panel). Moreover, Apatinib decreased VEGF secretion in a dose-dependent fashion in SGC-7901 (Figure 6B, left panel) and BGC-823 (Figure 6B, middle panel), but did not suppress VEGF secretion of MGC-803 cells (Figure 6B, right panel). We also confirmed that PLCγ1-ERK1/2 signaling pathway was responsible for the increment of cell proliferation induced by rhVEGF stimulation. We treated cells with DMSO or 500 nM of Apatinib overnight followed by 30min exposure to rhVEGF. Without Apatinib, rhVEGF treatment induced the phosphorylation of VEGFR2, PLCγ1, and ERK1/2 in SGC-7901 and BGC-823 cells. However, Apatinib diminished the rhVEGF induced phosphorylated VEGFR2, PLCγ1, and ERK1/2 to a sub-baseline level (Figure 6D). Taken together, Apatinib inhibited cell proliferation and VEGF secretion in cells that overexpressed VEGFR2, pVEGFR2, and VEGF.

### The efficacy of Apatinib on suppressing gastric cancer growth in xenograft tumor models

**Figure 7:**
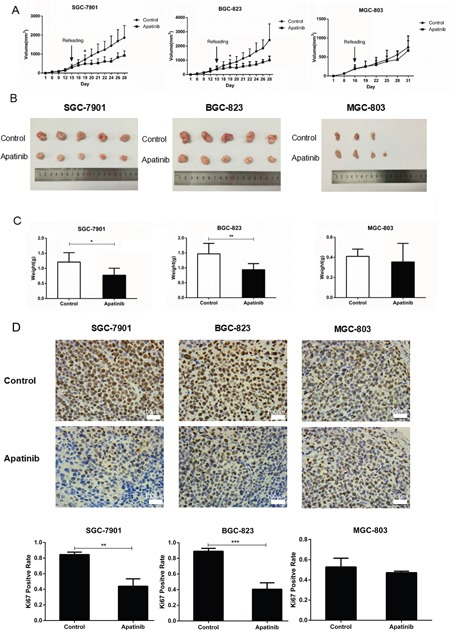
The efficacy of Apatinib on suppressing GC growth in xenograft tumor models **A**. In nude mouse xenografts of gastric cancer cells that overexpressed VEGFR2 and VEGF, Apatinib delays tumor growth. **B**. Apatinib decreased tumor volume in SGC-7901 and BGC-823 tumor models, but not in the MGC-803 model when mice were sacrificed. **C**. Apatinib-treated tumors decreased total tumor weights in SGC-7901 and BGC-823 tumor models, but not in the MGC-803 tumor model. **D**. Measured by IHC, Apatinib decreased the Ki67 positive rate of tumor cells in SGC-7901 and BGC-823 tumor models, but not in the MGC-803 tumor model. Mean±SEM, t-test, *P<0.05, **P<0.01, ***P<0.001.

To further explore the effect of Apatinib on gastric cancer cells with different activated VEGF signaling molecules, we established xenograft mouse tumor models by injecting SGC-7901, BGC-823, and MGC-803 cells subcutaneously into nude mice. When the mice developed a palpable mass (diameter≥0.5cm), they were treated with either Apatinib (50 mg/kg/day) or vehicle solution daily until sacrifice. The tumors formed by SGC-7901 or BGC-823 cell in mice which treated by Apatinib displayed a substantial delayed in growth after 4 days of treatment, as compared to the vehicle groups. The mean tumor volumes and tumor weights were significantly decreased between the vehicle and Apatinib-treated groups when the mice were sacrificed (Figure 7A&7B). However, in the xenograft tumor model formed by the MGC-803 cell which with lower activated VEGFR2 expression, the tumor growth, the mean tumor volumes, and tumor weight between the vehicle and Apatinib-treated groups did not show a significant difference (Figure 7A, 7B &7C). Using IHC staining for the cell proliferating index Ki-67, we found that Apatinib treatment reduced the number of Ki-67 positive cells in SGC-7901 and BGC-823 tumors, but not in the MGC-803 tumors (Figure 7D).

## DISCUSSION

In this study, we showed that gastric cancer cells produced VEGF which promoted tumor cell growth by activating VEGFR2. When activated by VEGF, the VEGFR2 were phosphorylated and trans-localized from cell membrane to cytoplasm and nucleus in gastric cancer cells. In addition, cell proliferation and secretion of VEGF decreased when VEGFR2 inhibited by NA or small molecular VEGFR2 inhibitors. We demonstrated that autocrine VEGF signaling promoted gastric cancer cell proliferation in a PLC-ERK1/2 dependent pathway. Administrating gastric cancer cells with Apatinib suppressed cell proliferation, decreased VEGF secretion, and reduced VEGF, pVEGFR2, PLC and ERK1/2 expression in a dose-dependent fashion. Furthermore, we demonstrated that gastric cancer cells that overexpressed VEGFR2 and VEGF were more sensitive to the growth suppressive effects of Apatinib. *In vivo*, treatment with Apatinib delayed VEGFR2 and VEGF overexpressing gastric cancer cell xenograft tumor growth and decreased tumor weight and volume.

It is well acknowledged that tumor-derived VEGF mediated the functions of endothelial cells in the tumor microenvironment [8]. However, little is known regarding the role of autocrine VEGF signaling in gastric cancer cells. There were increasing studies support the notion that VEGF, acting as a growth factor, has a direct effect on tumor cells and can sustain tumor cell growth in an angiogenesis-independent fashion [26]. VEGF could directly induce activation of VEGFR2 that lead to the activation of downstream signaling molecules in a cell proliferation pathway [27], suggesting that a functional VEGF autocrine loop may exist in GC. In endothelial cells, VEGF binding to VEGFR2 has been shown to induce its phosphorylation and translocation from cell surface to nucleus, where the VEGFR2 bind to the promoter to regulate its own transcription [9, 27]. Nuclear translocation of VEGFRs amplified angiogenesis and cell proliferation effects [26]. In this study, by Western blot and IF, we found that rhVEGF can activate VEGFR2 and induced its nuclear translocation, suggesting the existence of autocrine VEGF signaling loop in gastric cancer cells.

Autocrine VEGF signaling promotes tumor cell proliferation and viability through angiogenesis-independent pathways in several tumor types [23–24]. In gastric cancer cell lines, we found that treatment of rhVEGF increased cell viability, suggesting that exogenous VEGF signaling contributes to epithelial cell growth in gastric cancer cell lines. VEGFR2-NA competitively binds to VEGFR2 on the cell surface and inhibits the activation of VEGFR2. When treating cells with VEGFR2-NA for 24 hours, exogenous VEGF signaling did not increase cell viability. SU1498, the VEGFR2 inhibitor, can enter to the cytoplasm, binds to VEGFR2 fragments and inhibits the phosphorylation of VEGFR2. In this study, the treatment of SU1498 for 24 hours significantly inhibited cell proliferation, but exogenous VEGF signaling did not reverse the inhibitory effects of SU1498. Moreover, we found that the use of SU1498 decreased the production of VEGF. These results indicated that VEGF signaling promotes gastric cancer cell proliferation through an autocrine pathway. Therefore agents that target VEGFR2 could be used for prevention or treatment of GC.

To further explore the downstream molecular events in autocrine VEGF signaling, we stimulated gastric cancer cell with exogenous VEGF (rhVEGF) and observed the effects of a series of pharmacologic inhibitors. We found that VEGF signaling promotes gastric cancer cell proliferation by inducing the phosphorylation of PLC and ERK pathway. It is reported that VEGFR2 activated ERK directly through Ras, or indirectly through the PLC/PKC pathway [9, 28]. We observed that the inhibitor against PLC significantly reduced the expression of activated ERK, suggesting that VEGF activated ERK indirectly through PLC in gastric cancer cells. Furthermore, we confirmed that after blocking PLC, the production of VEGF decreased. These results suggested that inhibition of PLC decreased ERK activation, cell proliferation and VEGF secretion in gastric cancer cells.

Although Apatinib, a selective inhibitor of VEGFR2, has been approved by the FDA of the USA for the treatment of GC and confirmed to be a well effective treatment for gastric cancer patients [14], the molecular mechanism of Apatinib on the tumorigenesis of GC is still obscure. In this study, we found that Apatinib decreased cell proliferation and the production of VEGF in a dose-dependent fashion in gastric cancer cells which overexpressed VEGFR2 and VEGF, other than in low expression gastric cancer cell. Similarly, in the xenograft tumor models, treatment of Apatinib resulted in a significant delay in growth of tumor which formed by overexpressed other than low expressed VEGFR2 and VEGF gastric cancer cells. In summary, we demonstrated that Apatinib not only has anti-angiogenesis effects but also possesses substantial angiogenesis-independent effects. More importantly, we found that gastric cancer cells that overexpressed VEGFR2 and VEGF were more sensitive to the growth suppressive effects of Apatinib. These findings may enable better stratification of gastric cancer patients for clinical treatment decision.

## MATERIALS AND METHODS

### Cell culture and reagents

Five human gastric cancer cell lines (AGS, SGC-7901, BGC-823, MGC-803, and HGC-27) were obtained from the Cell Bank of Chinese Academy of Medical Science (Shanghai, China). Cell lines were cultured in RPMI-1640 medium containing 10% fetal bovine serum (Gibco, Life technologies, Thermo Fisher Scientific Inc.), penicillin (100 U/mL), and streptomycin (100 mg/mL). Recombinant VEGF was from R&D Systems (Minneapolis, MN). Apatinib (HengRui Medicine Co. LTD, Jiangsu, China), VEGF Receptor 2 neutralize antibody (R&D Systems, Minneapolis, MN.), SU1498 (Abcam plc, UK), U73122 (Sellock, Shanghai, China), were also used in our study (Supplementary Table 1).

### Western blot

Total protein, nuclear, cytoplasmic and membranous protein was extracted from cell culture according to instructions from respective protein extraction kits (Beibo, China). The protein concentration was quantified using an Enhanced BCA Protein Assay Kit. Fifty micrograms of protein from each sample were separated on 8% SDS-PAGE and transferred onto PVDF membrane (Immobilon-P, Millipore, US). The primary antibodies used were as follows: anti-VEGF, anti-VEGF Receptor 2, anti-phosphorylated VEGF Receptor 2, anti-PLCγ1, anti- phosphorylated PLCγ1, anti-ERK1/2, anti-phosphorylated ERK1/2, anti-GAPDH, anti-Tubulin, anti-Histone H3 (Cell Signaling Technology Inc., US, Supplementary Table 2). The blots were visualized using the enhanced chemiluminescence detection system (Tanon 5200, Shanghai, China). The experiments were repeated at least three times.

### Quantitative real-time polymerase chain reaction (qRT-PCR)

Total RNAs were extracted from cell pellets by using the RNA plus reagent kit (TaKaRa, Japan). Complementary DNA was synthesized using oligo^dT^ primers according to the protocol supplied with the Primer Script TM RT Reagent (TaKaRa, Japan) (Supplementary Table 3). Expression of VEGF was determined by quantitative real-time PCR using Power SYBR green PCR master mix (Applied Biosystems). The ratio of target gene and GAPDH mRNA expression in AGS cell was used as the normalization data. The experiments were repeated at least three times.

### Immunofluorescence staining

Cells were fixed with 4% paraformaldehyde for 20 minutes at room temperature. After washing with PBS, the Triton-100 was applied for cell membrane permeabilization for 10 minutes at room temperature. Cells were incubated with 5% bovine serum albumin to block the nonspecific binding sites. Primary antibodies were incubated with cells at 4°C overnight. After washing with PBS, the cells were then incubated with the secondary antibody for 1 hour at room temperature. 0.5μg/ml DAPI was used to stain cell nucleus. Cells were observed and photographs were taken in a fluorescence microscope. The experiments were repeated at least three times.

### Cell counting Kit 8 (CCK8) assay

Cell counting Kit-8 (CCK-8) assay was used to examine cell proliferation ability. In brief, cells were planted onto 96-well cell culture plates (Nest Biotechnology Co., Ltd, China) at a density of 2×10^3^ cells/well in 100μL CM or serum-free RIMP 1640 culture medium in the presence or absence of rhVEGF-NA, rhVEGF, VEGF receptor 2 neutralizing antibody, U73122, SU1498 and Apatinib for 24 hours. Then, 10μL CCK-8 reagents (Dongjido, Japan) were added to each well for 2 hours incubation at 37 °C according to the manufacturer's instructions. The absorbance was read at the wavelength of 450 nm in an automated plate reader. The experiments were repeated at least three times.

### Enzyme-linked immunosorbent assays (ELISA)

Gastric cancer cells were administrated with reagents for 4-6 hours, then washed with PBS 3 times and replenish with serum-free RIMP 1640 medium. Cells were incubated for 24 hours and media were collected for the VEGF concentration assay using an ELISA kit (Novex, Life technologies, Thermo Fisher Scientific Inc.). Briefly, a total of 100 μl/well condition medium and standard samples were added to the antibody-coated 96 well plates and incubated for 2 hours at room temperature, followed by addition of the biotin-conjugated polyclonal antibody specific for VEGF and incubation for 1 hour. Plates were then washed and incubated with avidin conjugated to HRP for 1 hour. The color was developed using TMB substrate, stopped by adding sulfuric acid and measured using a plate reader (infinite F50, Tecan) at a wavelength of 450 nm. The experiments were repeated at least three times.

### Immunohistochemistry

Tumor masses from xenograft experiments were processed using standard histological procedures, and tumors were evaluated for Ki67 staining. For IHC, deparaffinized sections were pretreated with 10 mM sodium citrate buffer for antigen unmasking (pH 6.0, boiling temperature, 30 min), blocked in normal serum (Vectastain ABC kit, Vector Laboratories, Inc. Burlingame, CA), incubated with primary antibodies at 4°C overnight, rinsed, and incubated with secondary antibody (Vectastain ABC kit). Signals were amplified using Vectastain ABC kit per manufacturer's instruction. Targeted protein was visualized using diaminobenzidine as substrate. The results were interpreted by two independent pathologists who were blinded to the specific diagnosis and prognosis for each case. Counts reflect the pathologists’ consensus. The intensity of IHC staining was estimated by a semi-quantitative scoring method.

### Collection of condition medium

The gastric cancer cells were grown in 15cm diameter cell culture dishes until around 80% confluency. The medium was aspirated off, and the monolayer was washed three times with PBS, once with serum-free RIMP-1640, and then replenished with serum-free RIMP-1640. After 48 hours incubation, the medium was collected, filtered and stored at -80°C until use.

### Apatinib treatment of xenograft tumors

SGC-7901, BGC-823, and MGC803 cells were inoculated at the right flank of nude mice. After developing a palpable mass, mice were randomized to either the Apatinib treatment or control group (N = 5 per group). Mice were administered a daily oral gavage with 50 mg/kg Apatinib [29] or vehicle-only solution. Tumor size and volume was calculated based an established method [25]. The animal experiment was approved by Medical Ethics Committee of the First Affiliated Hospital of Sun Yat-sen University.

### Statistical analysis

The SPSS ver. 18.0 (SPSS Inc., Chicago, IL) was used for analysis of the data. The results are expressed as mean ± SD. The t or t’ test was used for two-group data comparison. Multiple group data and multiple comparisons were analyzed by one-way ANOVA and LSD-t test. A P-value of less than 0.05 was considered to be statistically significant for all analyses.

## CONCLUSION

In conclusion, our study provided evidence of angiogenesis independent VEGF effects in gastric cancer cells. We demonstrated that VEGF, produced by gastric cancer cells, activates VEGFR2-PLC-ERK pathway that evokes cell proliferation and the production of VEGF. We also found that Apatinib, a small molecular VEGFR2 inhibitor, can inhibit cell proliferation and the secretion of VEGF. Gastric cancer cells that overexpressed VEGF and VEGFR2 were more sensitive to the treatment of Apatinib because of VEGF/VEGFR2 inhibition. These results suggested that agents targeting molecules involved in autocrine VEGF signaling might be used for prevention and treatment of GC.

## SUPPLEMENTARY MATERIALS TABLES



## Abbreviations

AIs: Angiogenesis inhibitors
CCK8: Cell counting Kit 8
CM: Condition medium
DMSO: Dimethyl Sulphoxide
ELISA: Enzyme-linked immunosorbent assays
ERK1/2: Extracellular signal-regulated kinase 1/2
GC: gastric cancer
IgG1: Immunoglobulin G1
NA: VEGF-neutralizing antibody
PLCγ1: Phosphoinositide phospholipase C γ1
qRT-PCR: Quantitative real-time polymerase chain reaction
rhVEGF: Recombination human VEGF
TKI: Tyrosine kinase inhibitor
VEGF: Vascular endothelial growth factor
VEGFRs: VEGF receptors
