# Supplementary material for: Autocrine VEGF signaling promotes cell proliferation through a PLC-dependent pathway and modulates Apatinib treatment efficacy in gastric cancer
Source: Oncotarget. 2017 Jan 3;8(7):11990–2002. doi: 10.18632/oncotarget.14467 (PMC5355320; doi:10.18632/oncotarget.14467)
Supplement: Supplementary file 1 [file oncotarget-08-11990-s001.pdf]

Autocrine VEGF signaling promotes cell proliferation through a PLC-dependent pathway and modulates Apatinib treatment efficacy in gastric cancer

SUPPLEMENTARY TABLES

Supplementary Table 1: Reagent for study

Reagent	Manufacturer
Recombinant VEGF	R&D Systems, Minneapolis, MN
Apatinib	HengRui Medicine Co. LTD, Jiangsu, China
VEGF Receptor 1 neutralize antibody	R&D Systems, Minneapolis, MN.
VEGF Receptor 2 neutralize antibody	R&D Systems, Minneapolis, MN.
SU1498	Abcam plc, UK
U73122	Sellock, Shanghai, China

Supplementary Table 2: Antibody for immunoblot

Antibody	Source information(cat#, vendor)	Dosage	Usage
VEGF	Ab1316, Abcam	1:1000 dilution	WB
VEGFR1	#2893, Cell Signaling	1:1000	WB
pVEGFR1	Ab62183, Abcam	1:1000	WB
VEGFR2	#2479, Cell Signaling	1:150 IF, 1:1000 WB	IF, WB
pVEGFR2 (pY1054-Y1059)	Ab5473, Abcam	1:100 IF, 1:1000 WB	IF, WB
PLC-γ1	#2822, Cell Signaling	1:1000	WB
pPLC-γ1	#2821, Cell Signaling	1:1000	WB
Erk1/2	#9102, Cell Signaling	1:1000	WB
pErk1/2	#9101, Cell Signaling	1:1000	WB
GAPDH	#97166, Cell Signaling	1:1000	WB
Tubulin	#2148, Cell Signaling	1:1000	WB
Histone H3	#4499, Cell Signaling	1:1000	WB
Ki67	#12202, Cell Signaling	1:400	IHC
CD31	#77699, Cell Signaling	1:100	IHC

Supplementary Table 3: Oligonucleotide primers

Primer	Sequence(5' to 3')	Location	Use
VEGF-5'	ATCTTCAAGCCATCCTGTGTGC	sense	Real-time PCR
VEGF-3'	CAAGGCCCACAGGGATTTTC	antisense	Real-time PCR
VEGFR1-5'	ATC ATT CCG AAG CAA GGT GTG	sense	Real-time PCR
VEGFR1-3'	AAA CCC ATT TGG CAC ATC TGT	antisense	Real-time PCR
VEGFR2-5'	AGGCAGCTCACAGTCCTAGAGC	sense	Real-time PCR
VEGFR2-3'	GTCTTTTCCTGGGCACCTTCTA	antisense	Real-time PCR
GAPDH-5'	TGCACCACCAACTGCTTAGC	sense	Real-time PCR
GAPDH-3'	GGCATGGACTGTGGTCATGAG	antisense	Real-time PCR
